# Analysis of drug-drug interactions in spontaneous adverse drug reaction reports from EudraVigilance focusing on psychiatric drugs and somatic medication

**DOI:** 10.1192/j.eurpsy.2024.715

**Published:** 2024-08-27

**Authors:** D. Dubrall, R. Weber, M. Boehme, M. Schmid, B. Sachs, C. Scholl

**Affiliations:** ^1^University Hospital of Bonn, 53127; ^2^Federal Institute for Drugs and Medical Devices, 53175; ^3^University Hospital RWTH Aachen, 52074, Germany

## Abstract

**Introduction:**

Patients with severe mental illnesses (SMI) are often exposed to polymedication. Additionally, the risk of somatic diseases is twice as high in patients with SMI as in individuals without a psychiatric disorder. Furthermore, drug–drug interactions (DDI) between psychiatric drugs and somatic medications are a well-known cause of adverse drug reactions (ADR).

**Objectives:**

The aim of this study was to analyse whether already known DDI related to psychiatric drugs and somatic medication still occur in everyday clinical practice.

**Methods:**

Therefore we identified all spontaneous ADR reports contained in the European ADR database EudraVigilance from Germany received between 01/2017 and 12/2021 reported for patients older than 17 years in which antidepressants, antipsychotics and mood stabilizers were reported as suspected/interacting (n= 9,665). ADR reports referring to intentional overdoses and suicide attempts were excluded (n= 9,276 left). We used the ABDATA drug information system in order to identify all potential DDI (pDDI). The identified reports with pDDI were then assessed individually to determine whether the respective DDI occurred.

**Results:**

1,271 reports with 728 potentially interacting drugs pairs related to psychiatric drugs and somatic medications with 2,655 pDDI were found. Restricted to potentially interacting drug pairs with more than 10 reports, (i) hyponatremias related to antidepressants and diuretics (n= 362, 32.6%), (ii) bleeding events related to selective serotonin reuptake inhibitors (SSRI) and platelet aggregation inhibitors, anticoagulants or non-steroidal antiinflammatory drugs (NSAID) (n= 295, 17.5%), and (iii) increased beta-blocker effects related to SSRIs and beta-blockers (n= 126, 11.3%) were the most frequently identified pDDI. After individual case assessment, in 33.3% (14/42), 23.7% (45/190) and 17.4% (8/46) of the reports bleeding events related to SSRIs and anticoagulants, SSRIs and platelet aggregation inhibitors and SSRIs and NSAIDs were reported. Hyponatremia was reported in 7.6% (22/289) of the reports related to antidepressants and diuretics and increased beta-blocker effects in 6.9% (8/116) of the reports related to SSRIs and beta-blockers.

**Conclusions:**

According to our analysis, well-known DDI still occur in the treatment of psychiatric patients with psychiatric drugs and somatic medication. Whenever possible, alternative drug combinations with a lower potential of DDIs may be considered or appropriate monitoring measures should be conducted.

**Disclosure of Interest:**

None Declared

